# An interpretable TimeMIL framework for fNIRS: differential diagnosis between schizophrenia and bipolar disorder

**DOI:** 10.3389/fpsyt.2026.1832221

**Published:** 2026-06-10

**Authors:** Zefeng Wang, Binbin Gong, Lan Mou, Qian Tan, Xinhua Shen, Ruifang Cui

**Affiliations:** 1 School of Information Engineering, Huzhou University, Huzhou, China; 2 Department of Neurosis and Psychosomatic Diseases, Huzhou Third Municipal Hospital, The Affiliated Hospital of Huzhou University, Huzhou, China; 3 School of Mental Health, Wenzhou Medical University, Wenzhou, China

**Keywords:** biomarker, bipolar disorder, deep learning, fNIRS, interpretability, schizophrenia, time series classification

## Abstract

**Background:**

Schizophrenia (SCZ) and bipolar disorder (BD) exhibit substantial overlap in clinical symptoms, making differential diagnosis a persistent challenge and leading to a high risk of misdiagnosis. Recently, functional near-infrared spectroscopy (fNIRS) during the verbal fluency task (VFT) has provided more objective neurofunctional markers for distinguishing between the two disorders than traditional behavioral scales. However, most existing studies have predominantly relied on conventional, hand-crafted feature analyses. A significant limitation of this approach is the scarcity of deep learning models tailored for high-dimensional fNIRS time-series data, coupled with a lack of methods for their interpretation.

**Methods:**

In this study, a time-aware multiple instance learning model (TimeMIL) was applied to fNIRS data (healthy controls [HC]: 52 participants, SCZ: 50 participants, BD: 67 participants) collected during VFT. This model captures global dependencies in multivariate, high-dimensional time series of fNIRS, which represents each fNIRS recording as a bag and automatically locates informative temporal segments via a learnable wavelet encoder and self-attention. Furthermore, an interpretability framework integrating GradientSHAP and Integrated Gradients was proposed to quantify segment-and region-level attributions. Model performance was evaluated using 5×5-fold cross-validation and an independent test set.

**Results:**

TimeMIL achieved 0.928 ± 0.016 accuracy and a macro-averaged AUC of 0.984 ± 0.007 for three-class classification, significantly outperforming other representative models, including one-dimensional convolutional neural networks (1D-CNNs), Transformers, and temporal convolutional networks (TCNs). Attribution analyses highlighted robust differences in the orbitofrontal cortex (OFC) across HC, SCZ, and BD, suggesting its potential as a candidate discriminative feature. The frontal pole cortex (FPC) and ventrolateral prefrontal cortex (VLPFC) exhibited SCZ-specific attribution patterns.

**Conclusion:**

This study represents the first application of TimeMIL to fNIRS-based psychiatric disease classification, accompanied by a dedicated interpretability framework that illuminates prefrontal dysfunction. By integrating high accuracy, robustness, and interpretability, the proposed interpretable TimeMIL framework facilitates development of objective, neuroimaging-based tools for early screening and differential diagnosis of major psychiatric disorders, pending further validation in clinical settings.

## 1 Introduction

The differential diagnosis between schizophrenia (SCZ) and bipolar disorder (BD) remains challenging due to extensive overlap in psychotic symptoms, which fuels misdiagnosis and delays effective treatment (1). To mitigate this, the field has increasingly sought objective neurobiological markers capable of discriminating these disorders with higher fidelity (2). Functional near-infrared spectroscopy (fNIRS), combined with verbal fluency tasks (VFT), offers a practical and sensitive avenue for probing prefrontal hemodynamics. Owing to its portability, temporal resolution, resistance to motion artifacts, and cost-effectiveness, fNIRS enables to capture brain activity differences under ecologically valid cognitive paradigms (3–5). Recent studies have shown that fNIRS-VFT paradigms yield neurofunctional indicators superior to traditional behavioral assessments for differentiating SCZ and BD, and machine learning has shown promise in detecting subtle hemodynamic and connectivity differences within prefrontal networks between the two disorders (6). Similar advances have also been found in major depressive disorder, where fNIRS-based machine learning classifiers have successfully identified spatiotemporal features and achieved high diagnostic accuracy, highlighting its potential as an objective biomarker across psychiatric conditions (7). Developing fNIRS-based objective aids is therefore clinically meaningful for improving diagnostic accuracy, reducing misclassification, and guiding personalized care (2, 3).

Despite encouraging results, two critical challenges remain. First, existing deep learning models for fNIRS rarely exploit the full temporal dynamics of high-dimensional time-series data, limiting their ability to capture complex neural signatures (8–10). Second, in clinical contexts, interpretability is key for elucidating the neurophysiological basis of model predictions, validating model behavior against established clinical knowledge, and ensuring transparency and trust in medical decision-making (11–15). Yet most models operate as “black boxesblue,” limiting interpretability in high-stakes medical decision-making. Thus, answering these questions is essential for developing the clinical translation of fNIRS-based deep learning systems toward reliable and interpretable diagnostic support.

Time series classification lies at the core of neuroimaging applications in brain–computer interfaces and neurological/psychiatric diagnosis. Deep learning approaches—most notably one-dimensional convolutional neural networks (1D-CNNs), Transformers, and temporal convolutional networks (TCNs)—have shown strong performance on sequential fNIRS and electroencephalogram (EEG) signals. 1D-CNNs were first used to classify fNIRS time series and distinguish BD during remission from healthy controls (HC), underscoring the potential of deep learning models in psychiatric contexts (3). Lee et al. (5) then combined deep 1D convolutions with channel embeddings and achieved 84.48% accuracy for depression classification, outperforming support vector machines (SVMs) and highlighting the ability of CNNs to capture prefrontal asymmetry. Meanwhile, transformer-based models leverage self-attention to learn long-range dependencies and have set new benchmarks. For example, Wang et al. (16) proposed fNIRS-T and its end-to-end variant fNIRS-PreT, surpassing CNNs and Long Short-Term Memory networks (LSTMs) across multiple public datasets. Liao et al. (17) further integrated CNN and Transformer within CT-Net, using dual-wavelength fusion to reach 98.05% and 77.61% accuracy on two datasets and validating feature–region consistency through interpretability. Compared with the previous two models, TCNs offer a long effective memory and parallelism, which outperform recurrent networks on diverse sequence tasks (18). Ding et al. (19) also introduced MASA-TCN with spatially aware convolutions and multi-anchor attention, achieving state-of-the-art EEG emotion regression/classification with robust ablation evidence.

Nevertheless, the aforementioned three models still did not capture global dependencies in multivariate, high-dimensional time series, just as fNIRS features possessed. Thus, a time-aware multiple instance learning framework (TimeMIL) was introduced, which combines learnable wavelet positional encoding with Transformer mechanisms to model temporal dynamics effectively, outperforming 26 state-of-the-art methods on 28 multivariate time series datasets (20). Our work would apply TimeMIL to fNIRS neuroimaging and benchmark it against three other representative models (1D-CNN, TCN, and Transformer).

Interpretability remains another critical axis in clinical contexts. Normally, *post-hoc* feature attribution methods—spanning Shapley-value extensions and Integrated Gradients (IG)—estimate the contribution of input features to model outputs to generate comprehensible attribution maps. Moreover, compensated IG was proposed to reduce computational cost without manual baselines while preserving explanation reliability in EEG and time series contexts (21). IG, combined with RNNs is also used to estimate effective brain connectivity and quantify nonlinear causal relations (22). Zhuo and Ge (23) introduced IG² to suppress attribution noise via iterative counterfactual paths. Most importantly, Turbé et al. (24) assessed multiple time series attribution methods (including SHAP and GradientSHAP), highlighting substantial method-dependent differences and pitfalls in standard evaluation practices. These findings motivate combining complementary attribution strategies for more robust, clinically meaningful explanations.

Against this backdrop, our work would construct an interpretable TimeMIL framework based on fNIRS signals for three-way differential diagnosis among healthy controls (HC), SCZ, and BD. We evaluated TimeMIL on prefrontal fNIRS signals during VFT from 169 participants, using a three-class setting with 5×5-fold cross-validation and an independent test set, and benchmarked it against 1D-CNN, TCN, and Transformer baselines. The framework leverages TimeMIL’s MIL paradigm—representing the entire time series as a bag and temporal segments as instances—together with learnable wavelet positional encoding and self-attention to capture sparse, psychopathology-related fNIRS hemodynamic dynamics. For *post-hoc* interpretability, we would integrate GradientSHAP and IG to identify salient temporal segments and prefrontal channels that most strongly drive decisions. This study first systematically applies TimeMIL to fNIRS-based psychiatric disease classification with a dedicated interpretability framework, aiming to deliver an accurate automated aid for SCZ–BD discrimination and to illuminate potential neurobiological markers that can guide clinical translation.

## 2 Materials and methods

**Figure 1:**
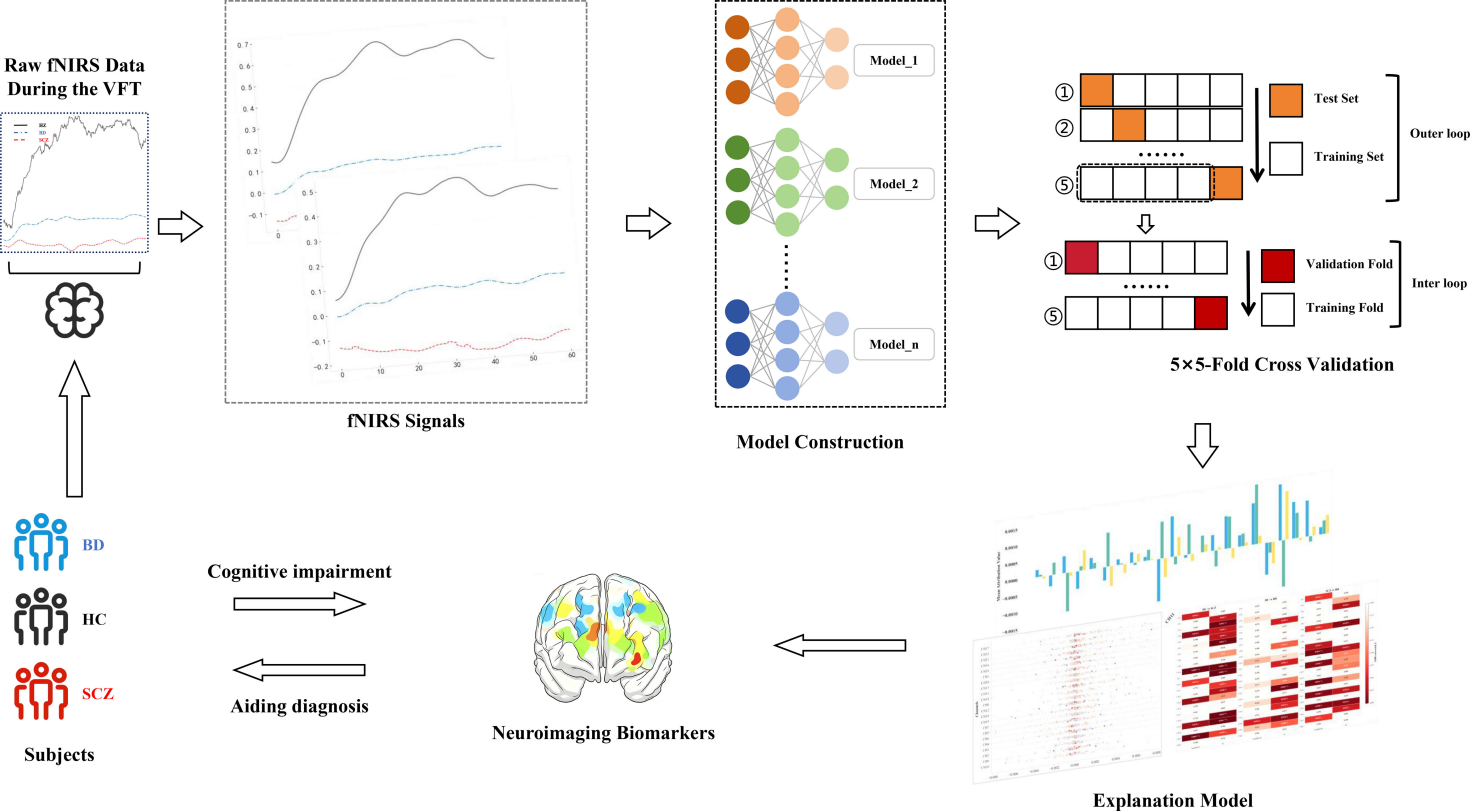
Overview of the TimeMIL-based pipeline for differential diagnosis among schizophrenia, bipolar disorder, and healthy controls using fNIRS during a verbal fluency task. Prefrontal fNIRS time series (22 channels, 600 time points) from 169 participants (SCZ = 50, BD = 67, HC = 52) are preprocessed (0.1 Hz lowpass and TDDR) and classified with TimeMIL; 1D-CNN, TCN, and Transformer serve as baselines. Posthoc interpretability combines Integrated Gradients and GradientSHAP to derive channel-level attributions, which are summarized and mapped to cortical regions for group comparisons.

The overall workflow of our study, from fNIRS data preprocessing to deep learning-based classification and *post-hoc* interpretability analysis, is schematically presented in **Figure 1**.

### 2.1 Dataset

Our dataset included prefrontal fNIRS recordings from 169 participants (50 with SCZ, 67 with BD, and 52 HC) that were recruited at the Third People’s Hospital of Huzhou between June 2022 and June 2025. All participants were required to complete the VFT adapted from Takizawa et al. (25), comprising a 30-s pre-task rest, a 60-s task, and a 70-s post-task rest. Subsequent analyses were restricted to the 60-s task period to isolate task-evoked prefrontal hemodynamic responses. Detailed demographics, inclusion/exclusion criteria, and full experimental procedures were provided in the **Supplementary Appendix** (**Supplementary Material**, **Supplementary Section 1**). This study was approved by the Institutional Review Board (IRB) of the Third People’s Hospital of Huzhou (Approval No. (2024) Lunshen No. 249), and written informed consent was obtained from all participants in accordance with the Declaration of Helsinki.

### 2.2 Data preprocessing

For each participant, raw oxygenated hemoglobin (Oxy-Hb) of fNIRS signals corresponding to the 60 s VFT task period were extracted for preprocessing. The selection of Oxy-Hb signals was based on their established superiority over Deoxy-Hb, specifically their greater sensitivity to cognitive activation and stronger correlation with the blood-oxygen-level-dependent (BOLD) signal measured in functional magnetic resonance imaging (fMRI) compared to Deoxy-Hb (26). A third-order Butterworth low-pass filter with a cutoff frequency of 0.1 Hz was applied to attenuate high-frequency physiological and instrumental noise (27), followed by Temporal Derivative Distribution Repair (TDDR) for correction of motion-induced artifacts and slow baseline drifts (28). After preprocessing, the dataset was partitioned at the participant level into a training set (80%) and an independent test set (20%) using stratified sampling to preserve the original SCZ/BD/HC class distribution. This pipeline was designed to enhance signal quality and cross-subject comparability, thereby providing a robust foundation for subsequent modeling; cross-validation procedures were conducted on the training set only, with the held-out test set reserved for final evaluation. Before entering the deep learning model, the shape of the dataset was (N = 169, C = 22, T = 600), where N is the total number of subjects, C is the number of channels, and T is the number of time points.

### 2.3 Deep learning model architectures

To validate the application of the fNIRS-based TimeMIL framework for psychiatric classification, the TimeMIL framework (20) is adopted as the primary model and benchmarked against representative temporal baselines, including 1D-CNN, Transformer, and TCN. Subsequently, the problem formulation and architectural designs are detailed below.

#### 2.3.1 TimeMIL

TimeMIL is often used for multivariate time series classification. It treats each participant’s time series as a bag and temporal segments as instances, enabling the identification of informative periods under weak supervision. Moreover, this model integrates multi-scale feature extraction with time-aware aggregation via learnable wavelet positional encoding and self-attention, which jointly capture sparse, delayed hemodynamic responses and long-range dependencies in fNIRS.

##### 2.3.1.1 *Problem* formulation

Let $X^{i}\in {\mathbb{R}}^{C\times T}$ denote the multichannel fNIRS sequence for participant i ( i=1,…,N). The observation at time point t is $x_{t}^{i}\in {\mathbb{R}}^{C}$, a C-dimensional vector across channels.

Each participant has a label $y^{i}\in \{0,1,2\}$ corresponding to HC, SCZ, and BD, respectively. For each class c, a binary MIL problem is defined with latent instance labels $y_{t,c}^{i}\in \{0,1\}$, under the standard MIL assumption (Equation 1):

(1) $$y_{c}^{i}=\underset{t}{\max}\{y_{t,c}^{i}\}$$

The model learns class scores $g_{c}(X^{i})$, which are implemented by a shared feature backbone and a 3-way softmax classifier. The output is a probability vector $p^{i}\in {\mathbb{R}}^{3}$, where $p^{i}[c]\approx g_{c}(X^{i})$. The predicted class is then (Equation 2):

(2) $${\hat{y}}^{i}=\text{arg }\underset{c}{\max}p^{i}[c]$$

##### 2.3.1.2 Model architecture

**Figure 2:**
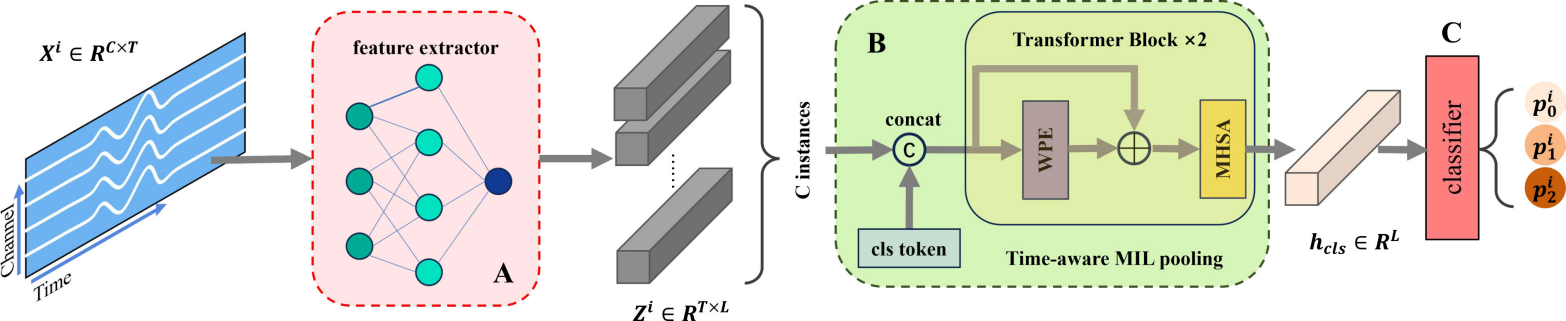
TimeMIL model pipeline. **(A)** a multi-scale 1D CNN maps *X_i_*to *Z_i_*. **(B)** Time-aware MIL pooling with a learnable wavelet positional term and tokenized Transformer pooling; the updated class token *h*_cls_ serves as the bag representation. C a classifier produces the three-class probability vector *y_i_*.

TimeMIL was used solely as the classification backbone for multivariate fNIRS in this study, rather than as model-intrinsic interpretability (interpretability analyses see Section X). The architecture comprises three modules: a multi-scale 1D CNN feature extractor, a time-aware MIL pooling module (a tokenized Transformer with learnable wavelet positional encoding), and a classifier. **Figure 2** illustrates the pipeline.

###### 2.3.1.2.1 Feature extractor

• Input: for participant *i*,


$$X^{i}\in {\mathbb{R}}^{C\times T}$$

• Output: per-time-point embeddings $Z^{i}=[z_{1}^{i},\ldots ,z_{T}^{i}]\in {\mathbb{R}}^{T\times L}$, where $z_{t}^{i}\in {\mathbb{R}}^{L}$.

• Computation: a multi-branch 1D CNN (Inception-style) captures local patterns at different temporal scales. ${\text{Conv}}_{k}(\cdot )$ denote 1D convolution with kernel size k, and ${\text{MaxPool}}_{3}(\cdot )$ denote max pooling with width 3 along time. For time point t (Equation 3):

(3) $$h_{t}=\text{concat }({\text{Conv}}_{10}{\left(X^{i}\right)}_{t},{\text{Conv}}_{20}{\left(X^{i}\right)}_{t},{\text{Conv}}_{40}{\left(X^{i}\right)}_{t},{\text{MaxPool}}_{3}{\left(X^{i}\right)}_{t})$$

(4) $$z_{t}^{i}=\text{ReLU }(h_{t}W_{f}+b_{f})$$

Here $W_{f}$ and $b_{f}$ are learnable parameters mapping the concatenated multi-scale features to L dimensions (e.g., L=128) (Equation 4). This produces one L-dimensional instance embedding per time point.

###### 2.3.1.2.2 Time-aware MIL pooling

This module aggregates instance *embeddings* into a bag-level representation while preserving temporal dependencies. Our work strictly follows the official *TimeMIL* implementation.

• Class token: a learnable class token $x_{\text{cls}}\in {\mathbb{R}}^{L}$ is *prepended* to the sequence (Equation 5):

(5) $$X_{\text{tok}}=[x_{\text{cls}};Z^{i}]\in {\mathbb{R}}^{(T+1)\times L}$$

• Learnable wavelet positional encoding (WPE): to encode temporal order and multi-scale time-frequency structure, *TimeMIL* injects a learnable wavelet-based positional term into the token sequence as implemented in the released code. Concretely, within each Transformer block, a wavelet positional bias $P\in {\mathbb{R}}^{(T+1)\times L}$ is computed from a bank of *learnlet-style* filters (initialized with a Mexican- hat/Ricker mother wavelet and learned scales/amplitudes) and added to $X_{\text{tok}}$ prior to attention (Equation 6):

(6) $$X_{\text{tok}}\leftarrow X_{\text{tok}}+P.$$

Thus, the positional term is applied to the whole token sequence (including the class token), following the official implementation.

• Transformer blocks and self-attention: multi-head self-attention (MHSA) is performed with standard projections (Equation 7)

(7) $$Q=X_{\text{tok}}W_{Q},\;K=X_{\text{tok}}W_{K},\;V=X_{\text{tok}}W_{V},$$

and an attention mask over padded positions (assigning large negative values to padded steps) (Equation 8):

(8) $$\text{Attn}(Q,K,V)=\text{softmax }(\frac{QK^{T}+M}{\sqrt{d_{k}}})V.$$

Each block uses *pre-norm LayerNorm*, residual connections, and a position-wise MLP. After several Transformer blocks, the updated class token *h*_cls_ ∈ R*^L^* is taken as the bag-level representation.

###### 2.3.1.2.3 Classifier

A multi-layer perceptron (MLP) maps the bag-level representation $h_{\text{cls}}$ (the updated class token from the TimeMIL pooling module) to a three-class probability distribution (Equations 9, 10):

(9) $$u^{i}=\text{ReLU }(W_{1}h_{\text{cls}}+b_{1})$$

(10) $$p^{i}=\text{softmax }(W_{2}u^{i}+b_{2})$$

yielding $p^{i}=\{p_{0}^{i},p_{1}^{i},p_{2}^{i}\}$ for HC, SCZ, and BD, respectively. Training uses standard cross-entropy over the three classes.

#### 2.3.2 Baseline models

Three baseline models were applied in this study: 1D-CNN, Transformer, and TCN. First, 1D-CNN adopted a three-block cascaded architecture in this study (3, 5). Each block consisted, in sequence, of a 1D convolution (kernel size = 7), batch normalization, a ReLU activation, and max pooling, designed to capture local temporal patterns in fNIRS time series. The network terminated with an adaptive average pooling layer over the temporal dimension, followed by dropout and a fully connected classifier. Batch normalization and dropout were included to stabilize optimization and improve generalization. Second, Transformer followed the standard encoder architecture with task-specific adjustments for time-series data (16, 17). A 1D convolutional input projection mapped the 22-channel fNIRS signals into a higher-dimensional temporal feature space. The encoder employed multi-head self-attention to capture global temporal dependencies and replaced the conventional LayerNorm with BatchNorm1d to better match the distributional characteristics of fNIRS sequences. Residual connections were retained to enhance training stability. This model targeted complex cross-time-step dependencies present in fNIRS signals. Third, TCN followed the architecture proposed by Bai et al. (18). Multiple temporal blocks were stacked; each block contained two 1D causal convolutions with an identical dilation rate to preserve temporal causality. Each convolution was followed by weight normalization, a ReLU activation, and dropout, and residual connections were incorporated to facilitate gradient flow. By increasing dilation rates exponentially across blocks, the receptive field expanded efficiently, enabling the model to capture multi-scale temporal dependencies in fNIRS time series. Its fully convolutional, parallelizable design yields efficient training performance. All three baseline models underwent the same hyperparameter optimization procedure as TimeMIL, including the same nested cross-validation framework and Bayesian optimization budget. The detailed search spaces and selected hyperparameters for each model are provided in **Supplementary Table 3**.

### 2.4 Model training and evaluation strategy

To comprehensively assess the aforementioned method, this work used a class-weighted cross-entropy loss to mitigate class imbalance and adopt the AdamW optimizer for training. A combination of regularization techniques further supported generalization, including dropout, batch normalization, early stopping, and learning-rate scheduling. All hyperparameters were selected via Bayesian optimization with the objective of minimizing the validation loss averaged across the five inner cross-validation folds, ensuring that the independent test set was never used for any model selection decision.

A 5×5 repeated stratified cross-validation framework was employed to validate TimeMIL and benchmark it against the 1D-CNN, Transformer, and TCN baselines. The procedure was as follows: (1) The dataset was split into an independent test set (20% of the data) and a development set (80%), where the test set was held out and not used for any training, validation, or hyperparameter tuning. (2) Within each of five independent repetitions, the development set was repartitioned via stratified 5-fold cross-validation, maintaining the original class distribution in each fold. In turn, one fold served as the validation set and the remaining four folds as the training set. (3) Validation loss was computed at the end of each epoch during training. Early stopping was triggered when the improvement in validation loss was less than 0.001 for 15 consecutive epochs (patience = 15, Δ_min_ = 0.001). The checkpoint with the lowest validation loss in each fold was retained as the optimal model for that fold. Critically, the independent test set remained completely isolated throughout the entire nested procedure. The five outer folds used mutually exclusive test partitions, collectively covering the entire dataset such that each subject was assigned to the test set exactly once per nested cross-validation run.

Evaluation strictly adhered to data isolation: every optimal model from cross-validation was evaluated on the independent test set. Accuracy, weighted precision, weighted F1-score, and macro-averaged Area Under the ROC Curve (macro-AUC) for each run were reported. In total, results from the 5 repetitions of the nested procedure were summarized as mean ± standard deviation. Finally, model comparison followed two criteria: (1) Comparison of average test performance across models, with macro-AUC as the primary metric. (2) Wilcoxon signed-rank tests (*p* = 0.05) were conducted to assess the statistical significance of performance differences between TimeMIL and each baseline, providing evidence for superiority and robustness.

### 2.5 Model interpretation methods

Two axiomatic attribution methods—Integrated Gradients (IG) and Gradient SHAP—were applied to the TimeMIL model to elucidate its decision-making mechanisms and identify neurobiological features relevant to mental-disorder classification. Triangulating attributions from these two complementary approaches helped quantify per-feature contributions and improved confidence in the identified fNIRS channels when their conclusions are consistent, thereby informing downstream biomarker discovery.

#### 2.5.1 Theoretical basis

##### 2.5.1.1 Target output (per class logit)

We attribute the *pre-softmax logit g_k_*(*x*) of the class of interest (e.g., the predicted class k), which mitigates *softmax-induced* gradient coupling.

##### 2.5.1.2 Integrated gradients

Given an input $x\in {\mathbb{R}}^{d}$ and a baseline $x^{\prime }$ of the same shape, the attribution for feature i is Equation 11:

(11) $${\text{IG}}_{i}\left(x;x^{\prime }\right)=\left(x_{i}-{x^{\prime }}_{i}\right)\int_{0}^{1}\frac{\partial g_{k}\left(x^{\prime }+\alpha \left(x-x^{\prime }\right)\right)}{\partial x_{i}}d\alpha$$

IG satisfies implementation *invariance* and sensitivity, and has completeness (Equation 12):

(12) $$\sum_{i=1}^{d}\text{IG}(x;x^{\prime })=g_{k}(x)-g_{k}(x^{\prime })$$

##### 2.5.1.3 Gradient SHAP

Gradient SHAP provides a smoothed, stochastic approximation to SHAP/Expected Gradients. Let D be a background (baseline) distribution over inputs, *α* ∼U(0, 1), $\epsilon ∼N(0,\sigma^{2}I)$. Gradient SHAP estimates *Shapley-style* attributions as Equation 13:

(13) $$\Phi_{i}^{\text{GS}}(x)=E_{x^{\prime }∼D,\alpha ∼U(0,1),\epsilon ∼N(0,\sigma^{2}I)}[(x_{i}-{x^{\prime }}_{i})\frac{\partial g_{k}(x^{\prime }+\alpha (x-x^{\prime })+\epsilon )}{\partial x_{i}}]$$

This method preserves *IG’s* implementation *invariance* and satisfies a completeness-in-expectation property (Equation 14):

(14) $$\sum_{i=1}^{d}{\text{Φ}}_{i}^{\text{GS}}(x)\approx g_{k}(x)-E_{x^{\prime }∼D}(g_{k}(x^{\prime }))$$

It does not guarantee exact SHAP Consistency or Local Accuracy for arbitrary deep models; these are approximated under suitable assumptions and adequate sampling.

#### 2.5.2 Method application

##### 2.5.2.1 Baselines

• IG: *x*^′^ is the per-channel global mean of the training set within the current fold, avoiding test leakage.

• Gradient SHAP: *x*^′^ is sampled from a background distribution *D* constructed from the current fold’s training data. Multiple baseline samples and *α* values are drawn, with small Gaussian noise *ϵ* for smoothing.

##### 2.5.2.2 Analysis unit (channel × time)

For each test sample, compute an attribution matrix $A\in {\mathbb{R}}^{C\times T}$ with both methods.

##### 2.5.2.3 Aggregation to channel importance

Signed importance per channel (Equation 15):

(15) $$\text{Mean}\;\text{Attribution}\;{\text{Value}}_{c}=\frac{1}{T}\sum_{t=1}^{T}A[c,t]$$

##### 2.5.2.4 Inter-group statistics

Group-wise mean channel attributions for SCZ, BD, and HC were computed. For each channel, pairwise group differences were tested using the Mann–Whitney U tests, with multiple comparisons controlled via False Discovery Rate (FDR) correction, and adjusted *p <* 0.05 was considered as significant.

## 3 Results

### 3.1 TimeMIL outperformed all competing baselines

The classification performance of four deep learning models was systematically evaluated on the three-class fNIRS task (SCZ, BD, and HC). As summarized in **Table 1**, the TimeMIL model achieved the highest mean performance across all evaluation metrics.

**Table 1:** Predictive performances of deep learning models on the test set.

Models	1DCNN	Transformer	TCN	TimeMIL
Accuracy	0.819 ± 0.011	0.752 ± 0.019	0.723 ± 0.022	0.928 ± 0.016
Macro-AUC	0.923 ± 0.008	0.872 ± 0.011	0.860 ± 0.010	0.984 ± 0.007
Precision				
HC	0.795 ± 0.023	0.745 ± 0.033	0.673 ± 0.054	0.939 ± 0.034
SCZ	0.878 ± 0.029	0.773 ± 0.054	0.864 ± 0.053	0.935 ± 0.029
BD	0.798 ± 0.032	0.742 ± 0.035	0.687 ± 0.044	0.917 ± 0.022
Weighted	0.821 ± 0.0101	0.752 ± 0.021	0.735 ± 0.018	0.929 ± 0.014
F1-score				
HC	0.797 ± 0.016	0.773 ± 0.022	0.673 ± 0.072	0.933 ± 0.019
SCZ	0.864 ± 0.013	0.799 ± 0.034	0.816 ± 0.050	0.919 ± 0.022
BD	0.803 ± 0.025	0.695 ± 0.036	0.691 ± 0.016	0.931 ± 0.016
Weighted	0.819 ± 0.011	0.750 ± 0.020	0.723 ± 0.025	0.928 ± 0.016

Values indicated mean ± SD (5×5-fold cross-validation). AUC, area under the ROC curve.

**Figure 3:**
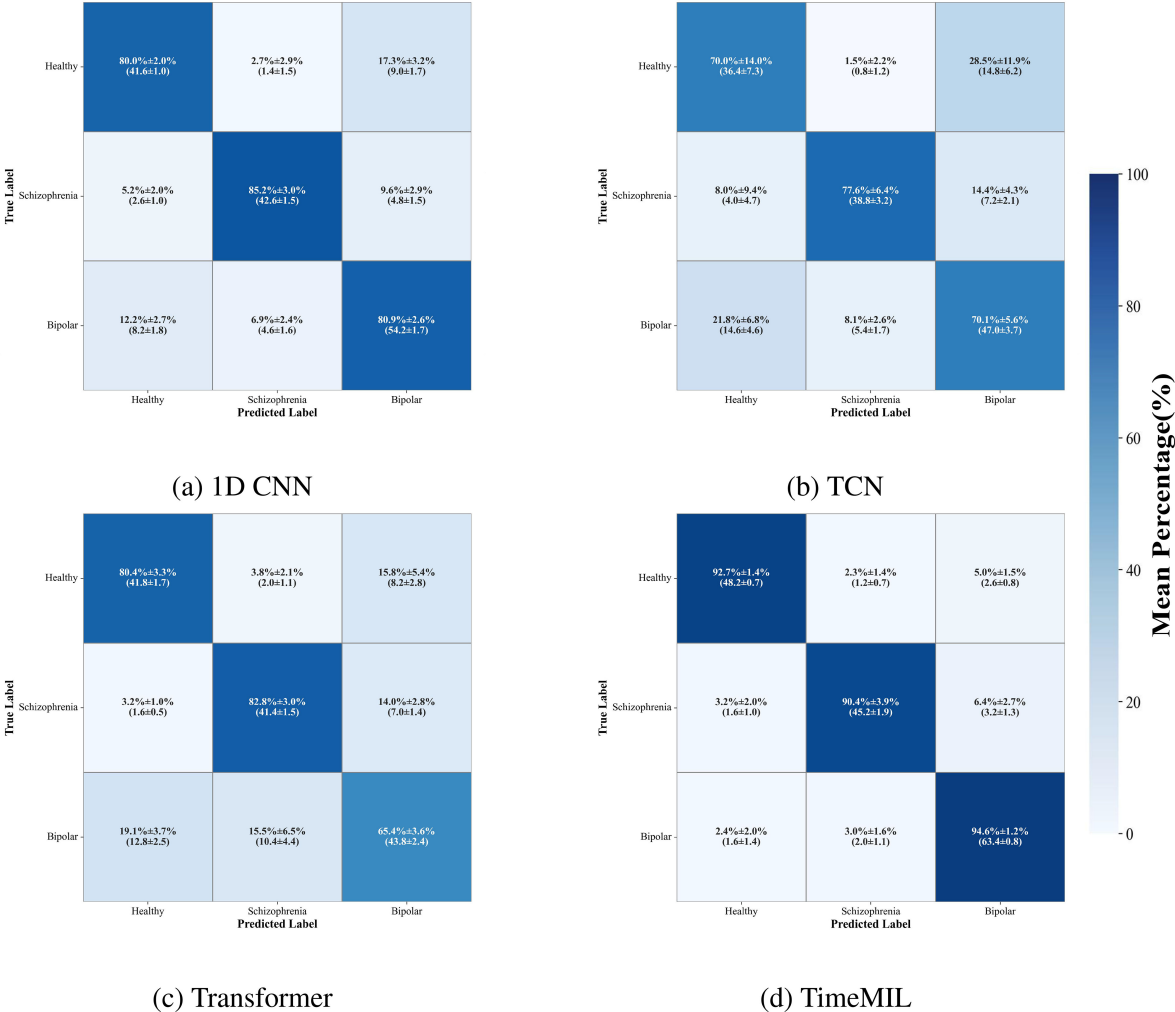
Normalized confusion matrices for the baseline models and TimeMIL. The subplots show the performance for **(a)** 1D-CNN, **(b)** TCN, **(c)** Transformer, and **(d)** TimeMIL. Values on the main diagonal represent the percentage of correctly classified samples for each class, ordered from top-to-bottom as Healthy Controls (HC), Schizophrenia (SCZ), and Bipolar Disorder (BD). Off-diagonal values indicate the percentage of misclassifications between classes.

**Figure 3** displays the confusion matrices for each model. For the baseline models shown in **Figures 3a–c**, the matrices show non-zero off-diagonal values, indicating misclassifications between the diagnostic groups. In contrast, the matrix for TimeMIL in **Figure 3d** shows values highly concentrated on the main diagonal, 280 with percentages for each class approaching 100% and minimal off-diagonal entries.

Paired Wilcoxon signed-rank tests confirmed that TimeMIL statistically outperformed all baseline models across all key metrics (all *p <* 0.001), with its macro-averaged AUC (0.984 ± 0.007) significantly surpassing that of 1D-CNN (0.923 ± 0.008), Transformer (0.872 ± 0.011), and TCN (0.860 ± 0.010).

Subject-level error profiling indicated that misclassifications were concentrated in a small subset of participants and were predominantly SCZ→BD (**Supplementary Table 2**).

### 3.2 Orbitofrontal cortex as a key region to distinguish the three groups

**Figure 4:**
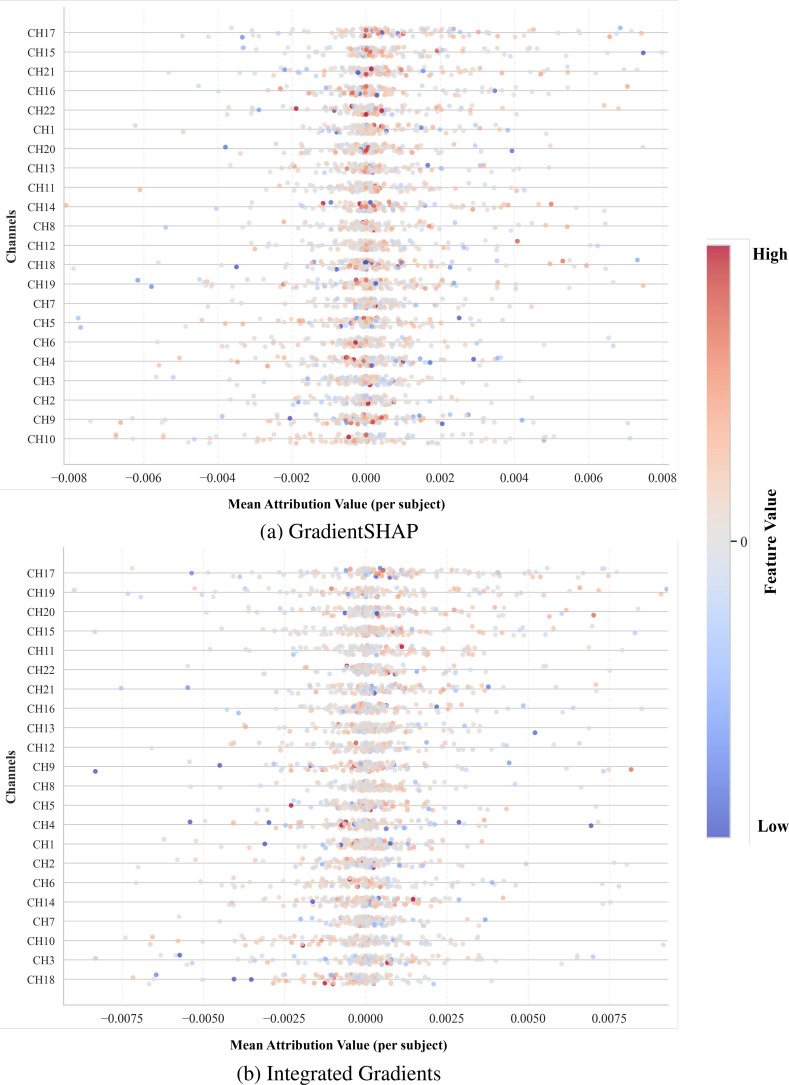
Swarm plots of *post hoc* attribution distributions across 22 fNIRS channels from the TimeMIL model. **(a)** GradientSHAP attributions; **(b)** Integrated Gradients attributions. Each dot represents a participant-level time-averaged attribution for a given channel; the x-axis denotes attribution magnitude (negative to positive), and the y-axis lists channel indices. Dot color encodes the corresponding feature value (blue = low, red = high).

**Figure 5:**
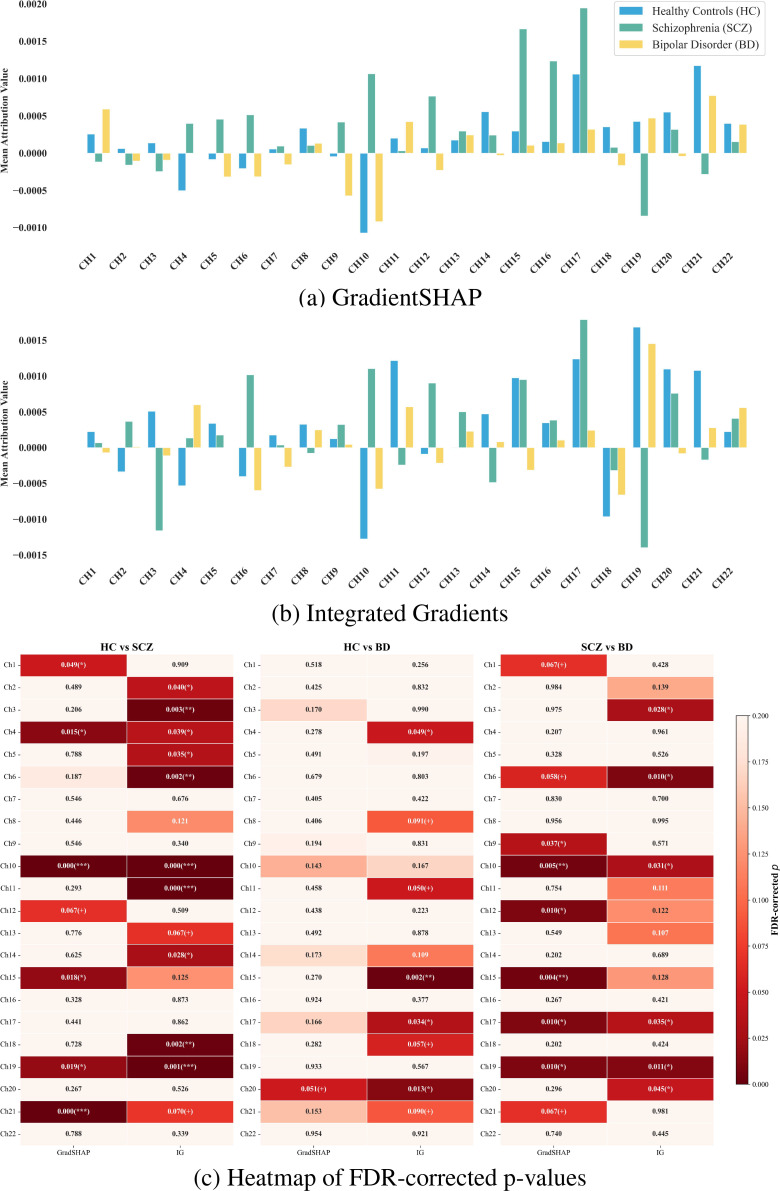
Group-wise mean attribution profiles and corresponding statistical comparisons. **(a)** Group-averaged, time-averaged attribution values from GradientSHAP. **(b)** Corresponding attribution values from Integrated Gradients. **(c)** Heatmap of FDR-corrected p-values from pairwise Mann–Whitney U tests comparing group attributions for each channel. Significance notation: *FDR-corrected *p <* 0.05; **p < 0.01; ***p < 0.001; + indicates raw *p <* 0.05 but failed FDR correction. Color intensity in the heatmap increases with smaller p-values.

Two gradient-based interpretability methods (i.e., GradientSHAP and IG) were employed to compute *post hoc* attributions for TimeMIL’s predictions, thereby elucidating its decision-making mechanism and pinpointing the cortical regions that most strongly influenced classification outcomes. As shown in **Figure 4**, the swarm plots from both attribution methods revealed marked heterogeneity in channel contributions. Notably, Channel 17 (orbitofrontal cortex, OFC) consistently exhibited the highest mean attribution under both methods, indicating a prominent role in the model’s discriminative process. To further investigate these channel-level contributions, **Figure 5** provides a comprehensive overview, depicting the group-averaged attribution values (Panels A and B) and the corresponding statistical results from pairwise Mann–Whitney U tests (Panel C). The key findings were as follows:

1. HC vs. SCZ: Significant differences in Channel 4 (dorsolateral prefrontal cortex, DLPFC), Channel 10 (frontopolar cortex, FPC), Channel 19 (ventrolateral prefrontal cortex, VLPFC), and Channel 21 (OFC); all FDR-corrected *p <* 0.05.
2. HC vs. BD: A marginally significant difference in Channel 20 (OFC) with GradientSHAP (uncorrected *p* = 0.002; FDR-corrected *p* = 0.051), and significant with IG (FDR-corrected *p <* 0.05).
3. SCZ vs. BD: Significant differences in Channel 6 (FPC), Channel 10 (FPC), Channel 17 (OFC), and Channel 19 (VLPFC); all FDR-corrected *p <* 0.05.

## 4 Discussion

This study systematically evaluated the application of the interpretable TimeMIL framework for the three-way classification of SCZ, BD, and HC using multivariate, high-dimensional fNIRS time series. Our findings establish that TimeMIL not only exhibits the strongest and most stable performance but also sets a new benchmark for this challenging diagnostic task. On the test set, TimeMIL achieved a state-of-the-art accuracy of 0.928 ± 0.016 and a macro-average AUC of 0.984 ± 0.007, significantly outperforming other representative temporal architectures, including 1D-CNN (5), TCN (18), and Transformer models (16, 17). This performance exceeds recent fNIRS classification benchmarks (29, 30), including the 98.05% AUC from CT-Net (17) and the 89.74% accuracy reported by Bayesian-tuned models (31). Critically, a deeper analysis of the confusion matrices (**Figure 3**) reveals the primary source of this superiority: TimeMIL’s exceptional ability to resolve the diagnostic ambiguity between SCZ and BD. While baseline models demonstrated considerable misclassification between the two patient groups, TimeMIL achieved near-perfect separation, particularly in correctly identifying BD patients. This specific achievement is of profound clinical relevance, directly addressing one of the most persistent challenges in psychiatry where misdiagnosis between SCZ and BD can lead to inappropriate treatments and worsened long-term outcomes. The results thus corroborate the broader conclusions of Chen et al. (20) regarding TimeMIL’s cross-domain robustness, while demonstrating its specific power in a clinically vital context.

The repeatedly misclassified individuals may represent borderline cases located along a phenotypic continuum spanning schizophrenia (SCZ), bipolar disorder (BD), and healthy controls (HC) (32). In such cases, the model may predominantly capture affective- and state-related dimensions (e.g., current mood state and task engagement) rather than perfectly aligning with categorical DSM diagnoses (33). Notably, the observation that several frequently misclassified participants exhibited subsequent diagnostic or symptomatic shifts at the 2-year follow-up suggests that these errors are unlikely to be purely random noise. Instead, misclassifications may be enriched in clinically unstable individuals or those with cross-spectrum features, and should therefore be interpreted as hypothesis-generating pending confirmation in larger longitudinal cohorts with standardized follow-up assessments.

The superior performance appears closely linked to the architectural fit between TimeMIL and the properties of fNIRS signals. By adopting a multi-instance learning paradigm, TimeMIL treated each time series as a “bag” of temporal segments, automatically prioritizing the most informative windows. This is exceptionally well-suited to the temporally sparse, task-evoked hemodynamic responses commonly observed during VFT, in which discriminative information is concentrated in specific moments rather than being uniformly distributed (34). Moreover, the combination of learnable wavelet positional encoding and self-attention enables unified modeling of long-range dependencies and local signal dynamics. The wavelet encoder adaptively decomposes fNIRS signals into multi-scale representations, enhancing sensitivity to the transient, task-related fluctuations that likely differ between SCZ and BD, while the self-attention mechanism captures global contextual interactions across both time and channels. Together, these components yielded a coherent and physiologically grounded representation of prefrontal hemodynamics, improving classification accuracy (35). In contrast, 1D-CNNs emphasize local feature extraction and are less adept at capturing extended temporal context; Transformers typically require larger datasets to fully realize their representational capacity; and TCNs, although expanding receptive fields via dilation, rely on fixed hierarchical structures less flexible for nonstationary dynamics (36). Thus, our findings demonstrated the TimeMIL framework was a superior choice for complex neural time series, in consonance with successful MIL applications in medical time-series analysis (37).

Moreover, interpretability analyses highlighted OFC as showing significant attribution differences across all pairwise group contrasts, revealing distinguish indicators among HC, SCZ, and BD. This result aligned with convergent neurobiological evidence. OFC showed structural and functional impairments in both SCZ and BD, representing a shared locus of vulnerability within affective and reward–motivational networks (38). Moreover, OFC cortical thickness and network connectivity are associated with amotivation—a negative-symptom dimension that spans the SCZ–BD spectrum (39). Recent review further indicated that the OFC contributed directly to higher-order adaptation and control in language/speech processing (40). These convergent findings plausibly account for the strong discriminative effect of the OFC observed across HC, SCZ, and BD. However, attribution differences in FPC and VLPFC appeared predominantly in contrasts involving SCZ, indicating greater disease specificity. Transcriptomic and functional imaging reports described widespread gene-expression abnormalities in the FPC of SCZ and stably reduced VLPFC activation during VFT (41), whereas BD was characterized by persistent VLPFC hyperactivation during manic states rather than depression states (42–44). We caution that these attribution results reflect model feature importance patterns and are correlational in nature, not causal. Independent neurophysiological validation would be required to establish clinical utility. In addition, DLPFC (channel 4 only) showed significance for HC vs. SCZ, consistent with the canonical view of DLPFC dysfunction as a hallmark of SCZ (45).

The interpretable TimeMIL framework based on fNIRS signals showed promise as a research-stage assistive tool for differential diagnosis. When paired with the noninvasive, low-cost, and portable attributes of fNIRS, the approach is well-aligned with current priorities in early detection of psychiatric disorders (46). Visualizable attribution matrices offer case-level transparency, addressing requirements set by regulations such as the EU GDPR (47) and initiatives like DARPA’s XAI (48), thereby facilitating clinical trust. The consistently informative channels (e.g., channel 20) may serve as candidate neuroimaging markers for stratifying disease subtypes, monitoring treatment response, and probing convergent mechanisms of prefrontal dysfunction. Moreover, given that psychological disorders are also prevalent among vulnerable populations such as the disabled elderly—who may suffer from depression, anxiety, or other mental health issues due to functional limitations—extending our fNIRS-based deep learning approach to this group could facilitate early detection and intervention, broadening the clinical utility of our findings.

However, several limitations merited careful consideration. First, a key limitation of this study is its single-center design with a modest sample size of 169 participants, which constrains the generalizability of the findings. Although robust cross-validation was used to mitigate overfitting, generalizability to broader populations and diverse settings should be confirmed via larger, multi-center external validation, echoing the trajectory of high-performing automated EEG systems such as SCORE-AI ()? Second, model development and validation were confined to VFT only; robustness across other cognitive paradigms (e.g., mental arithmetic, affective processing) and multimodal data (e.g., fMRI, EEG, genomics) remains to be established. Deployment on portable fNIRS platforms for real-time screening in point-of-care contexts is also urgently needed. Third, although clinical covariates (e.g., medication) were statistically controlled and TDDR was applied to attenuate systemic confounds, residual physiological influences (e.g., heart rate, respiration) cannot be fully excluded. Finally, the employed feature attribution techniques (GradientSHAP, IG) provide *post hoc* explanations that identify correlational—not strictly causal—associations.

## 5 Conclusions

This study developed and validated an interpretable TimeMIL framework for the three-way classification of SCZ, BD, and HC using multivariate, high-dimensional fNIRS time series. Leveraging architectures aligned with the temporally sparse structures of task-evoked hemodynamics, TimeMIL delivered outstanding and stable performance (0.928 ± 0.016; AUC 0.984 ± 0.007), outperforming established benchmark models. Interpretability analyses revealed OFC as a potential distinguishing biomarker among the three groups, while delineating more SCZ-specific involvement of the FPC and VLPFC. In sum, the interpretable TimeMIL framework offered a novel paradigm for complex neuro-temporal signals and showed clear promise as a research-stage assistive tool for differential diagnosis owing to its high performance and transparent rationale. Priorities for future work included external validation in larger multi-center cohorts, integration with multimodal data, and deployment on portable systems for real-time use, advancing this line of research from algorithmic innovation toward clinical validation.

## Data Availability

The raw data supporting the conclusions of this article will be made available by the authors, without undue reservation.
