# Supplementary material for: An interpretable TimeMIL framework for fNIRS: differential diagnosis between schizophrenia and bipolar disorder
Source: Front Psychiatry. 2026 Jun 10;17:1832221. doi: 10.3389/fpsyt.2026.1832221 (PMC13291161; doi:10.3389/fpsyt.2026.1832221)
Supplement: Supplementary file 1 [file DataSheet1.pdf]

Supplementary Material

1 PARTICIPANTS

A total of 117 patients were recruited from the Third People's Hospital of Huzhou between June 2022 and June 2025. Inclusion criteria were as follows: (1) a primary diagnosis of schizophrenia (SCZ) or bipolar disorder (BD) according to the Diagnostic and Statistical Manual of Mental Disorders, Fifth Edition (DSM-5), confirmed independently by two attending psychiatrists; (2) age between 18 and 60 years; (3) Han Chinese ethnicity; (4) right-handedness; (5) a minimum of primary school education; and (6) clinical stability with no changes to medication dosages for the two weeks preceding enrollment. All patients with BD were diagnosed with non-psychotic bipolar disorder.

Exclusion criteria included: (1) a history of any organic brain disease or other severe somatic illness; (2) receipt of electroconvulsive therapy (ECT) or repetitive transcranial magnetic stimulation (rTMS) within the past month; (3) a history of substance or alcohol abuse or dependence; and (4) current pregnancy or lactation.

Symptom severity was assessed using standardized clinical scales. The Positive and Negative Syndrome Scale (PANSS) was used to evaluate the presence and severity of psychotic symptoms in patients. The 24-item Hamilton Depression Rating Scale (HAMD-24) and the Young Mania Rating Scale (YMRS) were used to assess affective states. Current medications, including antipsychotics, antidepressants, and anticonvulsants, were documented and converted into defined daily doses (DDD) for standardization.

During the same period, 52 healthy controls (HC) were recruited from individuals undergoing routine health examinations at the same hospital. Inclusion criteria for the HC group were: (1) no history of systemic physical illness, no current or past psychiatric disorders, and no family history of psychiatric illness; and (2) no use of any psychotropic medication within the past year.

All participants provided written informed consent after receiving a complete description of the study. The study protocol was approved by the Institutional Review Board (IRB) of the Third People's Hospital of Huzhou **(Approval No. (2024) Lunshen No. 249)** and was conducted in accordance with the Declaration of Helsinki. The fNIRS data from all participants were used for the subsequent development and evaluation of the deep learning models.

2 FNIRS MEASUREMENT AND EXPERIMENTAL PARADIGM

2.1 Experimental Setup and Task Paradigm

fNIRS measurements were conducted in a quiet, sound-attenuated room. Participants were instructed to remain emotionally calm and minimize head movements throughout the experiment to ensure data quality. We employed a Chinese version of the verbal fluency task (VFT), adapted from the paradigm described by Takizawa et al.(Takizawa et al., 2008), to elicit prefrontal cortex activation. The task followed a block design Figure S1 consisting of: (1) a 30-second pre-task rest period, (2) a 60-second formal task period, and (3) a 70-second post-task rest period. During the rest periods, participants repeatedly vocalized the sequence "1-2-3-4-5" at a consistent volume. During the formal task period, they were instructed to generate as many words as possible starting with three common Chinese characters: (huā, flower), (jiāng, river), (hé, river), with 20 seconds allotted for each character. Standardized instructions were provided to

ensure all participants understood the procedure. To minimize artifacts, participants were asked to avoid head and neck movements, frequent blinking, and large mouth movements during the measurement. To isolate task-specific neural activity, all subsequent analyses were based on the fNIRS signals recorded during the 60-second task period.

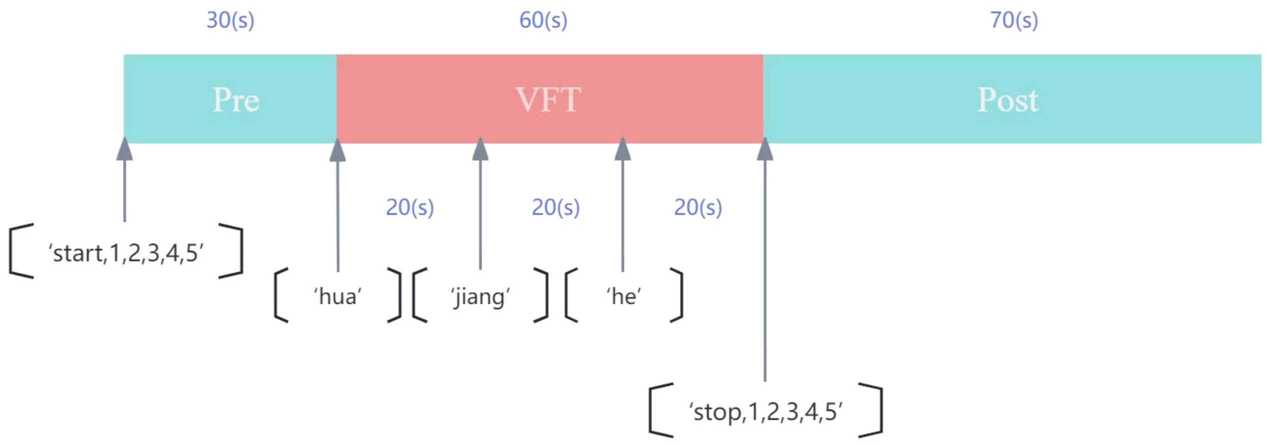

Figure S1. The verbal fluency task (VFT)

2.2 fNIRS Data Acquisition

Changes in oxygenated hemoglobin (Oxy-Hb) and deoxygenated hemoglobin (Deoxy-Hb) were measured using an ETG-one near-infrared spectroscopy system (Hitachi, Ltd., Japan). The system emits near-infrared light at two wavelengths (695 ± 20 nm and 830 ± 30 nm) from eight laser diodes and measures the reflected light using seven photodetectors. This configuration forms 22 measurement channels with a fixed inter-optode distance of 3 cm, providing a measurement depth of approximately 2-3 cm below the scalp, corresponding to the cortical surface (Okada and Delpy, 2003). The sampling rate was 10 Hz. The probes were arranged in a 3×5 grid placed over the forehead Figure S2. The middle optode of the bottom row was positioned at Fpz, according to the international 10-20 system. Channel locations were anatomically registered to Brodmann areas Table S1 based on validated probabilistic mapping from the 10-20 system (Jurcak et al., 2007). Relative changes in Oxy-Hb and Deoxy-Hb concentrations were calculated based on the modified Beer-Lambert law.

Table S1. Channel-to-Brain Region Mapping

Brain Region	Channels
Dorsolateral Prefrontal Cortex (DLPFC)	1, 2, 3, 4, 5, 9, 14, 18
Frontopolar Cortex (FPC)	6, 8, 10, 11, 12, 13
Orbitofrontal Cortex (OFC)	15, 17, 20, 21
Ventrolateral Prefrontal Cortex (VLPFC)	19, 22

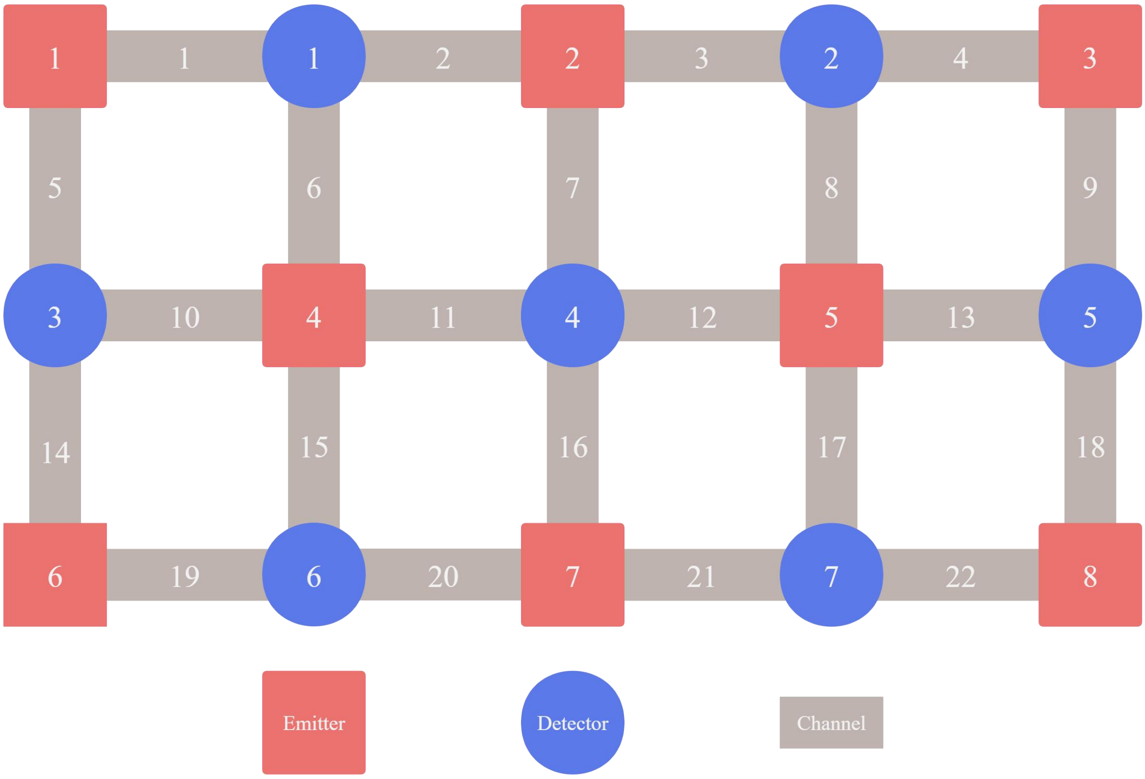

Figure S2. Light source, Detector and Channel layout

3 MISCLASSIFIED-INDIVIDUAL DEFINITION AND COUNTING SCHEME

Misclassification frequency was quantified at the individual level using the same evaluation framework as in the main text. We applied a repeated 5×5 cross-validation (CV) procedure, where each 5×5 CV run traverses the entire dataset and yields out-of-sample predictions on the held-out outer test fold only. Within one 5×5 CV run, each subject appears in the test set five times (once per repetition of the outer loop). We repeated the whole 5×5 CV procedure 20 times using different random seeds, resulting in 100 independent out-of-sample test predictions per subject (5 test appearances/run $\times$ 20 runs).

For each subject i with true label y_i, we aggregated the 100 predicted labels $\hat{y}_{i,j}$ and defined the total misclassification count as

$$M_i = \sum_{j=1}^{100} \mathbb{I}(\hat{y}_{i,j} \neq y_i)$$

where $\mathbb{I}(\cdot)$ denotes the indicator function. To summarize error directionality, we further recorded the most frequent incorrect predicted label (*top-1 misclassified to*) and its count, as well as the second most frequent incorrect label (*top-2 misclassified to*) and its count (when present). Subjects were ranked in descending order of M_i. “Frequently misclassified” individuals were operationally defined as those with a misclassification proportion $\geq 10\%$, i.e., $M_i \geq 10$ out of 100 predictions. For these individuals, we supplemented their 2-year follow-up information in the table (Table S2).

Table S2. Frequently misclassified individuals (misclassification proportion $\geq 10\%$).

ID	True	M_i	Top-1	Rate	2y status	Rehosp.
S001	SCZ	94	BD	0.936	depressive episode	NO
S002	HC	89	BD	0.865	NA	NA
S003	SCZ	80	BD	0.763	stable	NO
S004	SCZ	77	BD	0.701	depressive episode	NO
S005	HC	75	BD	0.947	NA	NA
S006	SCZ	64	BD	0.813	NA	NA
S007	BD	61	HC	0.672	hypomanic episode	YES
S008	BD	61	SCZ	0.803	stable	NO
S009	HC	60	BD	0.650	NA	NA
S010	HC	55	BD	0.836	NA	NA
S011	BD	47	HC	0.511	stable	NO
S012	HC	44	BD	0.523	NA	NA
S013	SCZ	35	HC	0.657	stable	NO
S014	SCZ	30	BD	0.667	stable	NO
S015	SCZ	26	BD	0.808	stable	NO
S016	SCZ	21	BD	0.571	stable	NO
S017	SCZ	20	BD	0.800	stable	NO
S018	HC	20	BD	0.900	NA	NA
S019	SCZ	20	BD	0.900	stable	NO
S020	SCZ	18	BD	1.000	stable	NO
S021	SCZ	14	BD	0.786	NA	NA
S022	BD	12	SCZ	1.000	stable	NO
S023	SCZ	11	BD	0.818	NA	NA

Note: Top-1 is the most frequent incorrect predicted label; Rate is the fraction of misclassifications assigned to Top-1; 2y status is the clinical status at 2-year follow-up; Rehosp. is any psychiatric rehospitalization within 2 years. NA indicates loss to follow-up. IDs were anonymized.

4 MODEL HYPERPARAMETERS AND SEARCH SPACES

Table S3 details the hyperparameter search spaces and the final selected values for all models evaluated in this study. All models were trained with the AdamW optimizer, a batch size of 128, a maximum of 100 epochs, and early stopping with a patience of 15 epochs based on validation loss. The same nested 5×5 cross-validation framework and validation metrics were used for hyperparameter selection across all models.All experiments were repeated with 20 different random seeds (1,2,3,...,20). The same set of seeds was used for all models to ensure comparability.

Table S4 reports the number of trainable parameters for each model as a reference for model capacity.

Table S3. Hyperparameter configurations and search spaces for all models.

Model	Parameter	Search Space	Selected
**1D-CNN**	Conv channels	[16,32,64] / [32,32,64] / [32,64,64]	[32, 32, 64]
	Kernel size	{3, 5, 7}	7
	Dropout rate	{0.2, 0.3, 0.5}	0.2
	Learning rate	$[5 \times 10^{-4}, 5 \times 10^{-3}, 5 \times 10^{-2}]$	5×10^{-2}
	Weight decay	$[1 \times 10^{-5}, 1 \times 10^{-3}, 1 \times 10^{-1}, 0]$	1×10^{-3}
**TCN**	Channels	[16,32,16] / [32,64,32] / [32,64,128]	[32, 64, 32]
	Kernel size	{2, 3, 5}	3
	Dropout rate	{0.2, 0.3, 0.5}	0.3
	Learning rate	$[5 \times 10^{-4}, 5 \times 10^{-3}, 5 \times 10^{-2}]$	5×10^{-3}
	Weight decay	$[1 \times 10^{-5}, 1 \times 10^{-3}, 1 \times 10^{-1}, 0]$	1×10^{-3}
**Transformer**	d_{model}	{32, 64, 128}	64
	nhead	{4, 8}	4
	nlayers	{2, 3, 4}	3
	dim_{FFN}	{32, 64, 128}	64
	Dropout rate	{0.2, 0.3, 0.5}	0.3
	Learning rate	$[5 \times 10^{-4}, 5 \times 10^{-3}, 5 \times 10^{-2}]$	5×10^{-4}
	Weight decay	$[1 \times 10^{-5}, 1 \times 10^{-3}, 1 \times 10^{-1}, 0]$	1×10^{-1}
**TimeMIL**	mDim	{64, 128, 256}	128
	Transformer layers	{4, 6, 8}	6
	Dropout rate	{0.2, 0.3, 0.5}	0.3
	Learning rate	$[5 \times 10^{-4}, 5 \times 10^{-3}, 5 \times 10^{-2}]$	5×10^{-2}
	Weight decay	$[1 \times 10^{-5}, 1 \times 10^{-3}, 1 \times 10^{-1}, 0]$	1×10^{-1}

Table S4. Trainable parameters per model.

Model	# Parameters
1D-CNN	27,011
TCN	38,371
Transformer	2,537,923
TimeMIL	745,172

REFERENCES

Jurcak, V., Tsuzuki, D., and Dan, I. (2007). 10/20, 10/10, and 10/5 systems revisited: their validity as relative head-surface-based positioning systems. *Neuroimage* 34, 1600–1611

Okada, E. and Delpy, D. T. (2003). Near-infrared light propagation in an adult head model. ii. effect of superficial tissue thickness on the sensitivity of the near-infrared spectroscopy signal. *Applied optics* 42, 2915–2921

Takizawa, R., Kasai, K., Kawakubo, Y., Marumo, K., Kawasaki, S., Yamasue, H., et al. (2008). Reduced frontopolar activation during verbal fluency task in schizophrenia: a multi-channel near-infrared spectroscopy study. *Schizophrenia research* 99, 250–262
